# Dynamic Estimation of Individual Exposure Levels to Air Pollution Using Trajectories Reconstructed from Mobile Phone Data

**DOI:** 10.3390/ijerph16224522

**Published:** 2019-11-15

**Authors:** Mingxiao Li, Song Gao, Feng Lu, Huan Tong, Hengcai Zhang

**Affiliations:** 1 State Key Laboratory of Resources and Environmental Information System, Institute of Geographic Sciences and Natural Resources Research, Chinese Academy of Sciences, Beijing 100101, China; limx@lreis.ac.cn (M.L.); luf@lreis.ac.cn (F.L.); 2 University of the Chinese Academy of Sciences, Beijing 100049, China; 3 Geospatial Data Science Lab, Department of Geography, University of Wisconsin-Madison, Madison, WI 53706, USA; song.gao@wisc.edu; 4 The Academy of Digital China, Fuzhou University, Fuzhou 350002, China; 5 Jiangsu Center for Collaborative Innovation in Geographical Information Resource Development and Application, Nanjing 210023, China; 6 UCL Institute for Environmental Design and Engineering, University College London, London WC1E 6BT, UK; huan.tong.18@ucl.ac.uk

**Keywords:** individual exposure estimation, air pollution, trajectory reconstruction, human mobility, mobile phone sensor

## Abstract

The spatiotemporal variability in air pollutant concentrations raises challenges in linking air pollution exposure to individual health outcomes. Thus, understanding the spatiotemporal patterns of human mobility plays an important role in air pollution epidemiology and health studies. With the advantages of massive users, wide spatial coverage and passive acquisition capability, mobile phone data have become an emerging data source for compiling exposure estimates. However, compared with air pollution monitoring data, the temporal granularity of mobile phone data is not high enough, which limits the performance of individual exposure estimation. To mitigate this problem, we present a novel method of estimating dynamic individual air pollution exposure levels using trajectories reconstructed from mobile phone data. Using the city of Shanghai as a case study, we compared three different types of exposure estimates using (1) reconstructed mobile phone trajectories, (2) recorded mobile phone trajectories, and (3) residential locations. The results demonstrate the necessity of trajectory reconstruction in exposure and health risk assessment. Additionally, we measure the potential health effects of air pollution from both individual and geographical perspectives. This helped reveal the temporal variations in individual exposures and the spatial distribution of residential areas with high exposure levels. The proposed method allows us to perform large-area and long-term exposure estimations for a large number of residents at a high spatiotemporal resolution, which helps support policy-driven environmental actions and reduce potential health risks.

## 1. Introduction

With the acceleration of urbanization and industrialization in the past few years, ubiquitous and unavoidable air pollution has become a widespread health problem in many developing countries [1,2]. Air pollution has been proven to be associated with higher health risk of respiratory infection, chronic obstructive pulmonary disease, stroke, heart disease, and lung cancer, among others [3,4,5]. According to the European Society of Cardiology, 8.8 million extra deaths can be linked to air pollution worldwide each year [6]. To solve this problem, a better understanding of the negative health effects of air pollution is a prerequisite, which leads to an increased demand of accurate individual air pollution exposure estimates in public health studies [7,8,9].

In the field of human exposure estimation, the importance of human mobility has long been recognized [7,10,11,12]. To address this problem, more detailed spatiotemporal human movement information is required. Although existing literatures have applied survey data [10,13,14,15,16], social media data [9,17,18,19,20] and mobile phone data [21,22,23,24,25] to record human movement behaviors, several limitations remain. As for the survey data-based methods, the cost of personal monitoring and sampling processes limits the number of samples, thereby reducing the persuasiveness of the estimation results [12,26]. In terms of social media data-based methods, representativeness is still problematic, as the data collected only reflects the individual movement characteristics of people who used the application at a specific time. 

**Figure 1:**
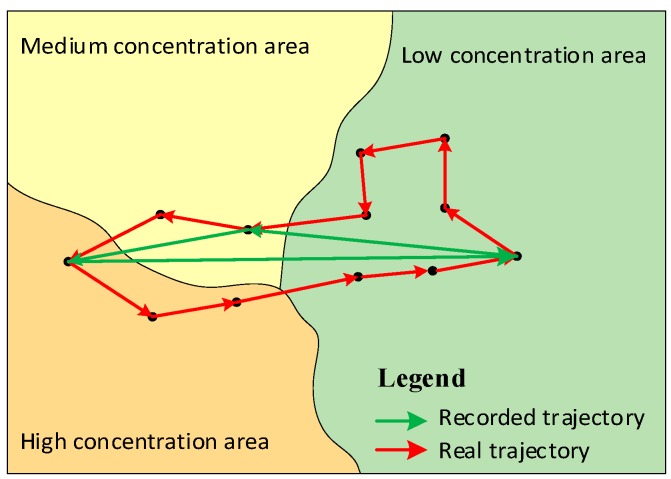
Illustration of the influence of the data sparsity problem in human movement data.

With the development of information and communication technology (ICT) and the ubiquity of mobile phones, mobile phone data have become an emerging dataset to measure human mobility. It has the advantages of massive users, wide spatial coverage and passive acquisition capability, which has the potential for long-term and well-represented exposure estimation. However, limited by the cost of data transmission and storage, most mobile phone data are only collected when the individual made a phone call or sent a message [27]. This inevitably limits the temporal granularity and regularity of mobile phone data, which introduces errors in exposure estimation [28,29]. As shown in Figure 1, the difference between real trajectory and recorded trajectory of an individual’s movement locations will inevitably result in incorrect estimation of the duration to which individuals are actually exposed to the areas with different air pollution concentrations. Therefore, improving the temporal granularity of mobile phone data is important for the exposure estimation.

In this paper, a novel method is proposed to estimate individuals’ air pollution spatiotemporal exposure levels using trajectories reconstructed from mobile phone data. The contributions of this paper are outlined as follows:

(1) We present a novel individual air pollution exposure estimate method. Our method mitigates the gap of spatiotemporal resolution between air pollution monitoring data and mobile phone data, which helps improve the accuracy and reliability of fine-scale air pollution exposure estimation.
(2) By comparing the three different types of exposure estimates using reconstructed mobile phone trajectories, recorded mobile phone trajectories, and residential locations, we demonstrate the necessity of trajectory reconstruction in exposure estimation.
(3) Using the city of Shanghai as a case study, we quantitatively analyzed the temporal variations in individual exposures and the spatial distribution of residential areas with high exposure levels using large-scale mobile phone data. It provides a more accurate and comprehensively scientific basis for policy-driven environmental actions and potential health risk reduction.

The rest of this paper is organized as follows. In Section 2, we review the research progress in related fields. The details of the proposed dynamic individual exposure estimation method are then presented in Section 3. The analysis of the case study and results are presented in Section 4; and finally, the conclusions and discussion on this study are presented in the final section.

## 2. Literature Review

### 2.1. Air Pollution Exposure Estimates

In the existing studies, researchers have attempted to measure air pollution exposure using various methods. According to the human mobility data that researchers have used, the related studies can be grouped into three categories: fixed-location-based methods, survey data-based methods, and mobile phone data-based methods. The fixed-location-based methods assume that individuals remain at a particular location and thus individual exposure can be calculated by measuring the air pollution concentration at their residence or workplace [30,31,32,33]. However, the spatiotemporal variability in air pollutant concentrations makes the human movement pattern a key factor in air pollution exposure estimation. That is, individual pollutant exposure estimates must be determined based on where the individual stays and how long the individual remains at each location. As neither an individual’s residence nor their workplace fully represents their daily movement pattern, the accuracies of these methods are not satisfactory.

To tackle this problem, researchers began to use survey data to record human movement behavior. The two most common techniques were manual sampling surveys [14,34] and Global Navigation Satellite System (GNSS)-enabled personal monitors [8,10,15,16]. For example, Yoo et al. (2015) used GNSS-equipped monitor data of 43 participants to demonstrate how an individual’s mobility affects personal exposure estimates. Similarly, Park and Kwan (2017) used 80 simulated daily movement trajectories to support the argument that ignoring human mobility patterns may lead to misleading results in exposure assessments. Although these techniques can provide fine-scale human movement data and additional personal information for further research, they have two limitations. First, the time requirements of the sampling process may cause the participants to become bored and result in recall bias [35]. Second, although the sampling method could be well designed, the cost of personal monitors and sampling processes limits the sample size and time period, making the results less persuasive.

The popularization of social media applications and location-based services provided a different way to track individual spatiotemporal activities. Compared with the survey data, the social media data enable us to collect human movement data with larger scale, longer term and broader spatial coverage [36]. Therefore, several researchers used social media data to estimate individual human exposure [9,17,18,19,20]. For instance, Song et al. (2019) combined sparse Weibo (a popular social media application in China) geotagged messages and remote sensing derived PM_2.5_ concentrations to perform monthly exposure estimations for 13 major cities in China. Yu et al. (2019) demonstrated the potential to estimate individual exposure with Google Maps location data on a minute level. However, the social media data-based method suffered from poor representativeness [37]. That is, the individual exposure inferred from these methods presents the exposure characteristics of active social media users rather than the whole population. The exposure risks of some demographic composition such as elderly and poor people tend to be misestimated since these people use the social media applications less often [38].

With recent advances in positioning techniques and the ubiquity of smart phones, mobile phone data, which can be collected without additional procedure and have wide stratified coverage, have been widely used for air pollution exposure estimation [9,21,23,25]. For example, Dewulf et al. (2016) proposed a method to estimate daily NO_2_ exposure using the mobility data collected from 5 million mobile phone users in Belgium. Yu et al. (2018) used mobile phone data to measure the influence of human movements on air pollutant exposure estimates. The use of mobile phone data enables us to measure individual exposure for a fairly high proportion of the entire human population over large areas and long periods. However, compared with the air pollution monitoring data, the temporal granularity of mobile phone data is not high enough, which limits the performance of individual exposure estimation [28,29]. Therefore, ways to improve the temporal granularity of mobile phone data has become a potential research hotspot in individual exposure estimation.

### 2.2. Trajectory Reconstruction from Mobile Phone Data

Trajectory reconstruction aims to approximate the locations of an individual that are missing in a mobile phone dataset. The basic methods of approximation rely on spatiotemporal interpolation [39,40,41]. It is assumed that an individual’s trajectory between two recorded points can be reconstructed using interpolation functions, such as the nearest function or a linear function. Therefore, the missing points can be approximated by the time intervals and distances between their contextual recorded points. These methods have been widely used for the reconstruction of intensive and continuous trajectories. However, the limited resolution of time intervals of mobile phone data and the complex individual movement patterns make these methods unsuitable for reconstructing trajectories from mobile phone data.

As vehicles are the main means of transport in the city [42], researchers have attempted to reconstruct missing points in low-frequency trajectories using map-matching-based methods. These methods assume that individuals’ trajectories are strictly dependent on road networks. Therefore, trajectory points can be snapped to road networks and missing points can be approximated based on movement characteristics along transportation networks such as velocity, acceleration, or travel time [43,44,45,46]. However, the precondition of these methods is questionable, as it ignores the fact that human movement behaviors in urban spaces can occur via the metro or on foot. Thus, the result of these methods is doubtful. 

With the development of machine learning and information and communications technology, researchers have focused on pattern mining methods. In these methods, the individual movement patterns are explored using historical trajectories and missing points are approximated by trained machine learning models [47,48,49]. However, limited by the high computational cost and the lack of efficient spatiotemporal proximity analysis methods [50], most pattern mining methods only use one individual’s trajectories or a single trajectory segment to approximate missing points. This leaves only a few amounts of data that can be used for model training, which leads to model overfitting and poor generalization issues. Thus, using trajectories of individuals with similar movement patterns as training data has become an efficient way to enhance reconstruction performance [51].

## 3. Methodology

**Figure 2:**
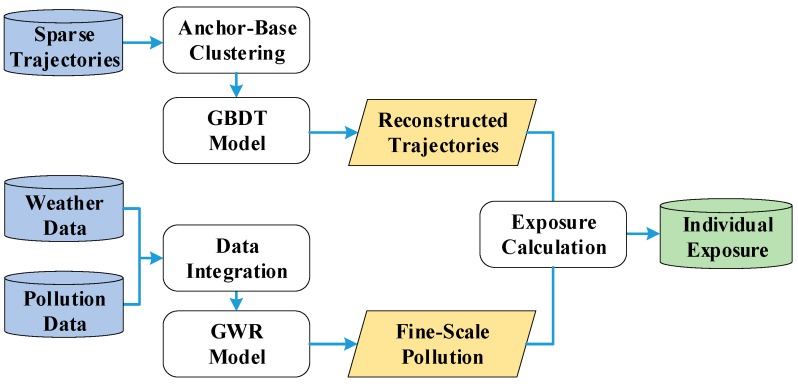
The workflow of the dynamic individual exposure estimation method.

The workflow of our method is presented in Figure 2, which includes three parts. First, we introduce a trajectory reconstruction algorithm to mitigate the gap of spatiotemporal resolution between air pollution monitoring data and mobile phone data. Then, we explain our algorithm to estimate air pollution concentrations on a fine scale. Finally, we show how dynamic individual exposure levels are calculated by combining the results of fine-scale air pollution concentration measurements and reconstructed trajectories. 

### 3.1. Anchor-Point Based Trajectory Reconstruction Algorithm

Due to the characteristic of low acquisition cost and wide spatiotemporal coverage, mobile phone data have been widely used in air pollution exposure estimation. However, due to the gap of spatiotemporal resolution between air pollution monitoring data and mobile phone data, trajectory reconstruction is important. Thus, an anchor-point-based trajectory reconstruction algorithm is presented in this subsection to grasp highly dynamic human movement trajectories with corresponding time.

#### 3.1.1. Anchor-Point-Based Clustering

As discussed above, using trajectories of individuals with similar movement patterns as training data has become an efficient way to enhance reconstruction performance. Since anchor points can summarize the key locations of individuals’ movement behaviors well, it becomes an efficient means of understanding the similarities among massive numbers of individuals. 

In our research, the anchor point is defined as an area where an individual visit more frequently than a specified number of times [52]. In this process, the number of records on each location in a personal dataset is first calculated. Then, the location with the highest record number is selected and merged with all adjacent locations within a distance threshold α as an examinee [53]. Next, the location with the second highest record number is selected and the same process was repeated until all locations in the personal mobile phone data had been traversed. Finally, the total record number is calculated for each examinee, and the examinees with record number exceeding frequency threshold β of their total record number are detected as anchor points and projected onto a subdistrict. Thus, an individual’s trajectories can be generalized as a collection of anchor points.

**Figure 3:**
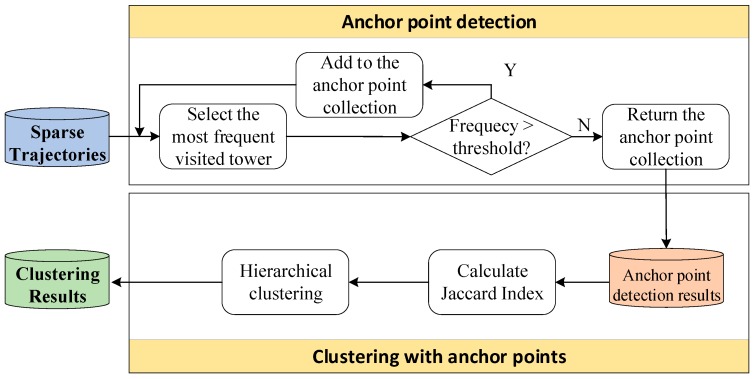
Flowchart of the anchor-point-based clustering method.

After that, based on the respective anchor point collections of individuals, the similarity matrix between the individuals could be calculated with the Jaccard index [54]. Then, the hierarchical clustering method [55] is used to divide the individuals into a series of groups. With this process, the movement patterns of the individuals in each group are similar. Therefore, the model can be trained with all the trajectories in each group rather than using an individual’s trajectories, which helps solve the overfitting problem. The flowchart of the anchor-point-based clustering method is presented in Figure 3.

Note that the choices of distance threshold and frequency threshold affect the clustering result. In terms of the distance threshold, we choose 500 m as the distance threshold for two reasons. First, in our case study area, the average distance between two adjacent locations in the mobile phone datasets is about 240 m. The threshold of 500 m could help reduce signal oscillation problem. Moreover, this threshold has been widely applied in existing studies of anchor point detection and human mobility [53,56]. As for the frequency threshold, it mainly refers to the proportion of the total number of records recorded at key activity locations such as residences and workplaces. Choosing a large threshold will result in the number of user identification anchor points being too small, which makes the user movement characteristics not fully summarized. On the contrary, a small threshold will recognize the unimportant locations as anchor points, which results in additional computational costs. For the data we used in the case study, we found that most of the users have about 20% of their recorded locations at residence or workplace (i.e., the anchor points with the most records during nighttime or daytime hours, respectively), therefore the frequency threshold is set to 20%. It is worth noting that such thresholds might vary in different cities.

#### 3.1.2. Reconstruction of Clustered Trajectories Using a Gradient Boosting Decision Tree Model

After dividing the individuals into a series of clusters, we apply a gradient boosting decision tree (GBDT) model to each cluster to infer the location of a target point. GBDT is an ensemble machine learning model with classification and regression tree (CART) as weak learners [57]. It generates a series of decision trees using the gradient boosting method and the result are determined via the summation of the weak learners [58]. A weak learner indicates a classifier that is only weakly correlated with the true classification [59]. To approximate the location of a missing point, a few characteristics were chosen to describe an individual’s movement patterns. The characteristics are selected based on two aspects. First, the following features were chosen to learn the movement pattern from historical trajectory segments, including the locations and times of contextual points $p_{n-1}$ ($x_{n-1},y_{n-1},\;t_{n-1}$), $p_{n+1}$ ($x_{n+1}$, $y_{n+1}$, $t_{n-1}$), and the time of the target point $p_{n}$ $\left(t_{n}\right)$; $p_{n-1}$, $p_{n}$, and $p_{n+1}$ denote three consecutive points along a trajectory that were resampled with different time intervals. Second, the radius of gyration (ROG) and the Shannon entropy (Ent) of each individual were chosen to describe their general movement patterns [24,29], which are defined as follows:

(1) ROGk=1o∑j=1l(ptj→−ptc→)

(2) $$Ent_{k}=-\overset{o}{\underset{j=1}{\sum }}p\left(j\right)\log_{2}p\left(j\right)$$

where o indicates the number of locations recorded in the individual’s dataset, $\vec{pt_{j}}$ indicates the $j^{\mathrm{th}}$ location, $\vec{pt_{c}}$ is the barycenter of all the records (ptc→=1o∑j=1optj→), and p(j) is the visit frequency of the $j^{\mathrm{th}}$ location. 

**Figure 4:**
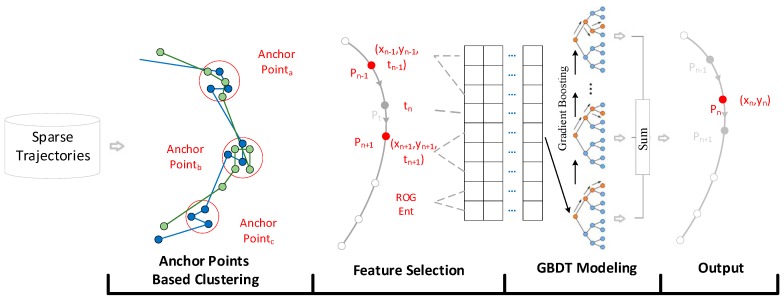
Architecture of the trajectory reconstruction algorithm.

Therefore, each training vector record can be represented as $\left[x_{n-1},y_{n-1},t_{n-1},t_{n},x_{n+1},y_{n+1},t_{n+1},ROG_{k},Ent_{k}\right]$ and the label data can be represented as [$x_{n},y_{n}$]. The architecture of the trajectory reconstruction algorithm is shown in Figure 4. More technical details of the proposed trajectory reconstruction algorithm can be found in the paper [51]. All the mobile phone trajectories were reconstructed with one-hour intervals using the trained model (i.e., 00:00, 01:00, …, 23:00, UTC + 8) to conform with the frequency of air pollution monitoring.

### 3.2. Estimation of Spatiotemporal Concentrations of Air Pollution

Estimating air pollution concentrations is the second step in quantifying individual air pollution exposure. As a practical technique, extending the ordinary linear regression framework, the geographically weighted regression (GWR) model [60] can examine the spatial variations and nonstationary aspects of a continuous surface of parameters at the local scale and is widely used in air pollution estimation [9,61]. In this study, we applied the GWR model to estimate air pollution concentrations based on the meteorological attributes of the surrounding area and used atmospheric particulate matter with a diameter of fewer than 2.5 micrometers (PM_2.5_) as a case study. It is a major type of air pollution whose concentration shows seasonal patterns and temporal variability [62] and which can be deposited deep in the lungs through simple respiration, causing an increase in respiratory and cardiovascular diseases [63].

**Figure 5:**
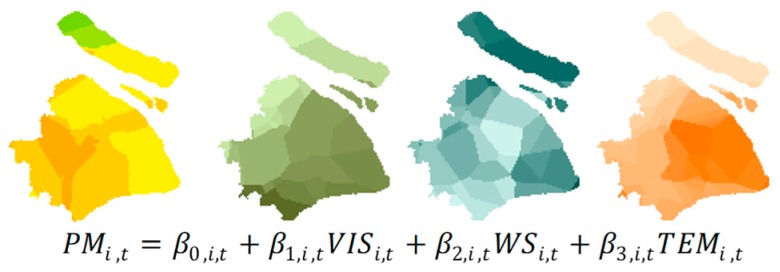
Illustration of the geographically weighted regression (GWR) model.

Considering the differences in geographic locations between air pollution monitoring stations and meteorological stations, the datasets need to be made consistent with respect to their spatial domains. Thus, an ordinary Kriging model [64] is first used to estimate meteorological variables with a spatial granularity of 1 km. Then, the meteorological observations within 1 km were averaged and assigned to the corresponding air pollution monitoring station to lessen the spatial mismatch bias. After that, a series of GWR models are developed as shown in Figure 5:

where i and t denote the corresponding location ID and time, $PM_{i,t}$ denotes the PM_2.5_ concentration (μg/m^3^), $VIS_{i,t}$ denotes the horizontal visibility (m), $WS_{i,t}$ denotes the wind speed (m/s), $TEM_{i,t}$ denotes the air temperature (°C), and $\beta_{0,i,t}$, $\beta_{1,i,t}$, $\beta_{2,i,t}$, and $\beta_{3,i,t}$ denote the regression coefficients of the corresponding features. In this study, as the temporal granularities of air pollution monitoring data and meteorological observation data are 1 hour, in total, 168 GWR models were trained to approximate the air pollution concentration distribution of each hour during one week. Finally, the finalized GWR models are ascertained based on model performance denoted by fitting the highest coefficient of determination ($R^{2}$) and the lowest Akaike information criterion (AIC) value. With the finalized modes, the optimal coefficients are used to approximate the air pollution concentration distribution in the study area with a spatial granularity of 1 km.

It is worth noting that there are two reasons for our choice of a spatial granularity of 1 km. First, since air pollution exposure estimation is a typical study of the environmental influences on individual behaviors, its spatiotemporal resolution is limited by the lower resolution data of the human movement data and the air pollution concentration data. Considering that the positioning errors of the mobile phone data in urban spaces range from 100 m to 1000 m [65,66], we chose a 1-km grid to divide the space. Second, this spatial granularity has also been widely used in previous studies to estimate pollutant concentrations and individual exposures to them [10].

### 3.3. Dynamic Individual Exposure Calculation

Since individuals’ locations and corresponding air pollution concentrations vary in both space and time, we propose an algorithm to incorporate dynamic individual locations, the spatiotemporal variation in air pollution concentrations, and the microenvironment effect to estimate the dynamic individual exposure as follows:

(3) $$Exp_{j}=\sum_{t=1}^{T}\sum_{n=1}^{N}AP_{i,t}*ME_{i,n,t}*TP_{i,n,t}$$

where $Exp_{j}$ denotes the dynamic exposure of individual j, i and t denote the corresponding location ID and time, N denotes the total number of microenvironments experienced by individual j within a specified temporal window (e.g., an hour) and n denotes the $n^{th}$ microenvironment, $AP_{i,t}$ denotes the outdoor air pollution concentration, $ME_{i,n,t}$ denotes the ratio of the air pollution concentration in the $n^{th}$ microenvironment to the outdoor air pollution concentration, and $TP_{i,n,t}$ denotes the percentage of time that the individual stayed in the $n^{th}$ microenvironment.

However, research on the impact of air pollution concentrations on the microenvironment has remained at the stage of qualitative analysis of small sample data [10,14]. The temporal resolution requirements and costly monitors make it impossible to acquire accurate observations on a large scale. Moreover, many factors, such as ventilation, air conditioning, smoking, and cooking, are independent of the outdoor environment, but can influence an individual’s microenvironment [67,68,69]. That is, individual exposure can be quite different at the same time and in the same area [35]. Thus, we define air pollution exposure as the outdoor exposure level in this study and simplify the ideal algorithm in Equation (3) to suit the estimation of large-scale dynamic individual exposure using the following equation:

(4) $$Exp_{j}^{\prime }=\sum_{t=1}^{T}\sum_{n=1}^{N}AP_{i,t}$$

where $Exp_{j}^{\prime }$ denotes the cumulative individual exposure from the simplified algorithm by only considering outdoor air pollution concentration and individual exposure duration.

## 4. Case Study

### 4.1. Data

#### 4.1.1. Mobile Phone Data

**Figure 6:**
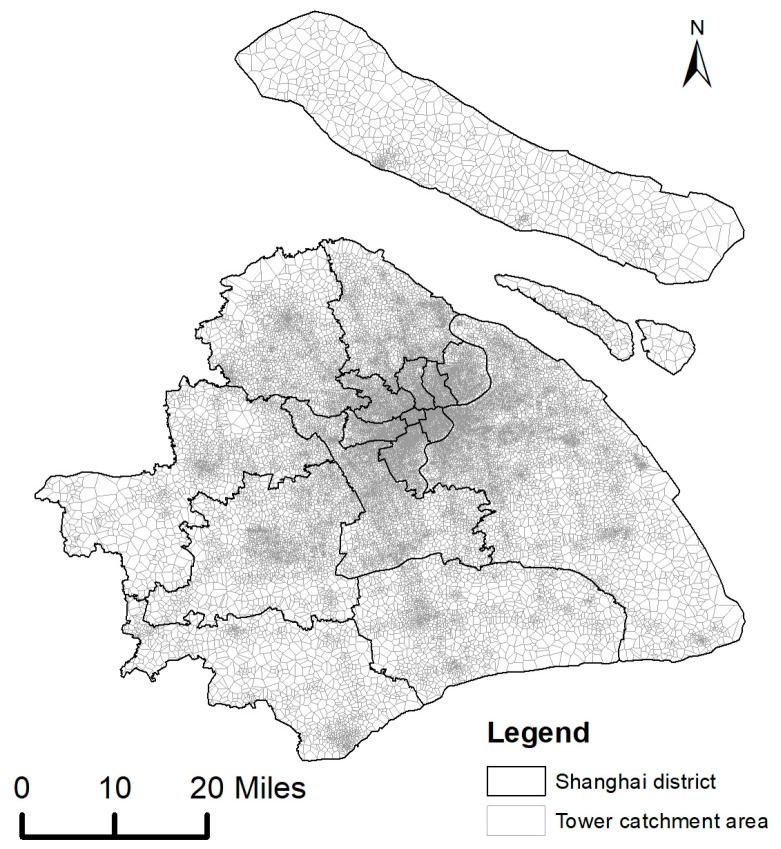
Map of the study area.

**Figure 7:**
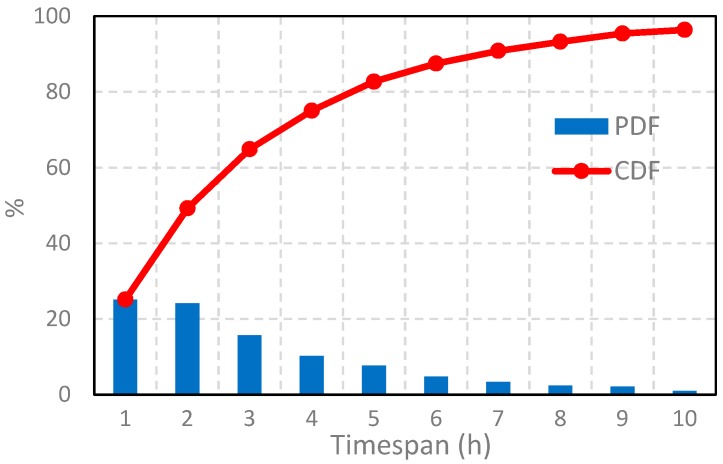
The distributions of the probability density function (PDF) and the cumulative distribution function (CDF) of time intervals between two adjacent call detail records in the recorded mobile phone data.

The mobile phone data we used were provided anonymously by a mobile network operator through a joint research cooperation. It records the location trajectories of over a million individuals for seven consecutive days in the city of Shanghai, China. A map of case study area is shown in Figure 6. According to the Shanghai Statistical Yearbook 2018, the operator accounts for about 56% of the city’s residents and is widely distributed among all strata of society (Shanghai municipal statistics bureau 2019). The dataset contained call detail records (CDRs, i.e., phone calls and text message) and actively generated records (i.e., regular updates, periodic updates, and cellular handovers). To decrease the signal oscillation problem, a repetition suppression algorithm [70] was used for data preprocessing. Table 1 shows an instance of one individual’s trajectory data. It is worth noting that none of the personal identifiable information (i.e., name, gender, phone number) were provided to protect the individuals’ privacy. In addition, the locations of the trajectory points were projected to the locations of ambient cell phone towers. In other words, there is still a gap between the user’s actual location and the projected trajectory points, which is about 100 m–1000 m on average in the dataset dependent on the specific area (e.g., downtown vs. suburbs). The distribution of time intervals between two adjacent call detail records is shown in Figure 7.

#### 4.1.2. Environmental Data

Hourly ground-station PM_2.5_ concentration data (in μm/m^3^) were collected from the data center of the Ministry of Environmental Protection of the People’s Republic of China (http://datacenter.mee.gov.cn) and the World Air Quality Index project (http://aqicn.org/city/shanghai/). Moreover, ground-station meteorological variables, including horizontal visibility, air temperature, and surface wind speed were collected from the National Meteorological Information Center (http://data.cma.cn/). The instances of ground-station PM_2.5_ Concentration Data and meteorological Data are shown in Table 2 and Table 3.

In line with the mobile phone data, environmental data were modeled during the corresponding days. To mitigate the estimation biases and improve spatial interpolation accuracy at marginal areas of the study area, the environmental data from two neighboring provinces, Zhejiang and Jiangsu, were also adopted for air pollution concentration estimation to add sufficient surrounding information for the locations at the boundary of study areas. Thus, the data from a total of 178 monitoring stations and 150 meteorological stations were collected for monitoring ambient air quality.

### 4.2. Spatiotemporal Variability in PM_2.5_ Concentration

**Figure 8:**
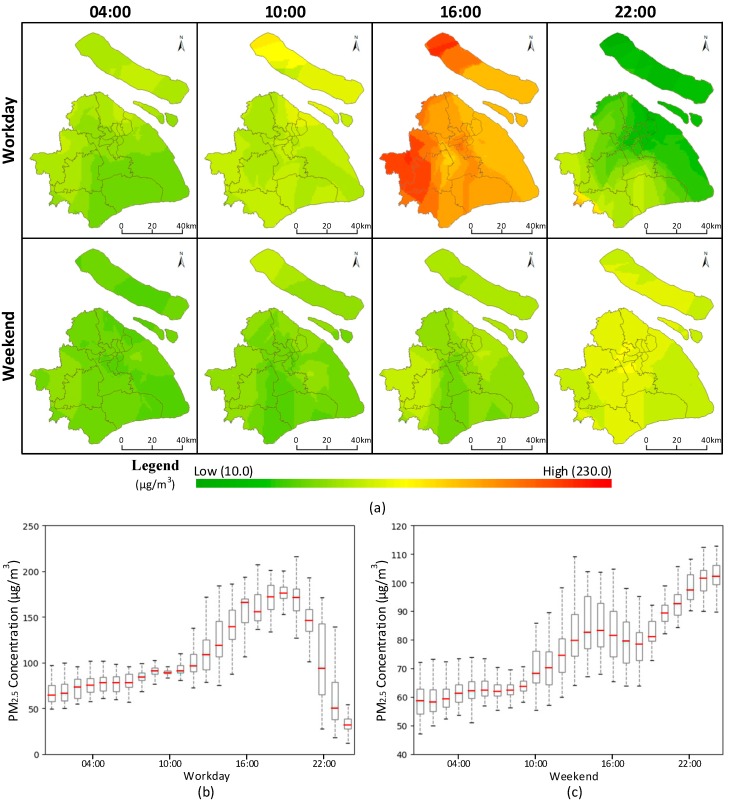
Different facets of PM_2.5_ concentration. (**a**) Hourly maps of PM_2.5_ concentration distribution on a workday and a weekend. (**b**) Temporal variation of PM_2.5_ concentration on a workday. (**c**) Temporal variation of PM_2.5_ concentration on a weekend.

The spatiotemporal PM_2.5_ concentration variation is an important component of the individual exposure estimation. Figure 8 shows examples of the extracted data (collected on a workday and a weekend) on selected hourly maps (e.g., 04:00, 10:00, 16:00, and 22:00) and the temporal variation curves for PM_2.5_ concentrations, which were approximated by GWR models mentioned in Section 3.2. It clearly reveals the spatiotemporal variations in PM_2.5_ concentrations in the study area, suggesting that the results of individual exposure estimates are directly related to individual movement behaviors across locations and time. Therefore, temporal mismatch between human movement data and PM_2.5_ concentrations will inevitably introduce errors into the individual exposure estimation. Thus, the requirement of consistent recording times of human movement data and PM_2.5_ concentration data makes trajectory reconstruction a key preprocessing step for more accurate individual exposure estimation.

The fitting results of the GWR models were evaluated using the metrics of R-squared and root mean square error (RMSE). The average R-squared between the predicted and observed PM_2.5_ concentrations is 0.81, and the average RMSE is 21.18 μg/m^3^, both of which are acceptable for dynamic air pollution exposure estimation [9].

### 4.3. Performance Evaluation of the Trajectory Reconstruction Algorithm

**Figure 9:**
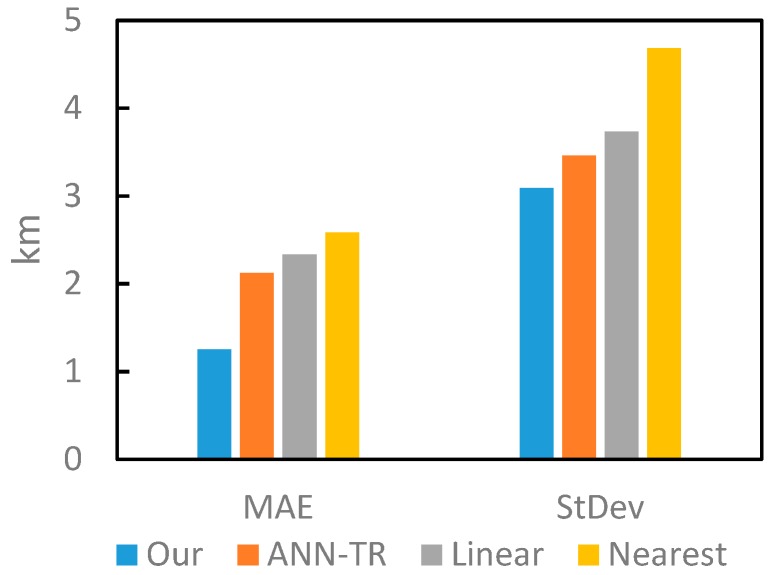
Comparison of the proposed method with baseline approaches using the indicators of mean absolute error (MAE) and StDev.

In our proposed method, the accuracy of air pollution exposure estimation directly depends on the accuracy of trajectory reconstruction. Thus, we compared the performance of our proposed trajectory reconstruction algorithm with other existing methods. To verify the performance of the proposed trajectory reconstruction algorithm, all the CDR data were extracted and used as “recorded data” (about 34% of the total records) and the actively generated records were used as “missing data” (about 66% of the total records). Thus, the average performance of reconstruction algorithms could be evaluated using the mean absolute error (MAE) between the reconstructed locations and the actively generated record locations, and the stability of the reconstruction algorithms could be evaluated using the standard deviation of the errors. In addition, one artificial neural network-based reconstruction algorithm, ANN-TR [49], and two most widely used algorithms, nearest interpolation and linear interpolation [40], were chosen as baselines. The performance comparison of these algorithms is shown in Figure 9.

As shown in Figure 9, our proposed algorithm shows lower reconstruction error and better robustness (with lower StDev) than baselines, which proves the superiority of our proposed method over these existing ones. More technical details about different trajectory reconstruction evaluation results can be found in [51].

### 4.4. Comparison with Existing Exposure Estimate Methods

To quantitatively analyze how the gap of spatiotemporal resolution between human movement data and air pollution monitoring data affects individual exposure estimates, three types of individual exposure estimates were obtained by (1) using reconstructed mobile phone trajectories for exposure estimation (hereafter, TR-EE); (2) using recorded mobile phone trajectories for exposure estimation (hereafter, REC-EE); and (3) using static locations (home location) for exposure estimation (hereafter, SL-EE). As the statistical results of individual exposure levels do not conform to a normal distribution, the K-S test [71] was applied to access the differences among these three types of exposure estimates, which are shown in Table 4. The results show that all the estimated pairs have larger K-S statistics than the expected values under null hypothesis and with very low *p*-values, which indicates that the exposure estimates of TR-EE are significantly different from those of the other two.

**Figure 10:**
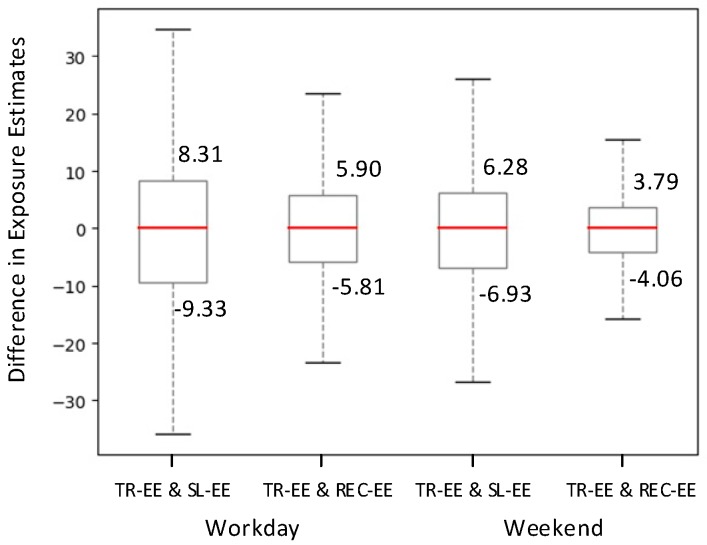
Box plots of the differences in each pair of exposure estimates on a workday and a weekend.

Figure 10 shows the difference between TR-EE and the other two types of exposure estimates with a box plot. The lengths of the interquartile range boxes of the TR-EE & SL-EE cases are larger than those of the boxes for the TR-EE & REC-EE cases on both weekdays and weekends. This means that the exposure estimation errors for the both middle half of the individuals and all the individuals increase when the home locations are used for exposure estimation than when the recorded mobile phone trajectories are used. In addition, we can see that the median values (the red lines in each box) of the four cases are approximately zero and the boxes are basically symmetrical around the median line. This indicates that the results of exposure estimation can be either overestimated or underestimated when the recorded mobile phone trajectories or static locations are used. It demonstrates that neither the home location nor the recorded mobile phone data can fully represent individuals’ movement behaviors when estimating dynamic individual air pollution exposure. Therefore, the necessity of trajectory reconstruction when using mobile phone data to estimate individual exposure levels is clarified. Further, due to varying patterns of individual movement behaviors on weekends (with the average Shannon entropy of 1.40) compared to that on weekdays (with the average Shannon entropy of 1.45), the exposure estimation errors on weekends are slightly different from those on workdays.

### 4.5. Potential Health Effects

Based on the ambient air quality standards proposed by the Chinese National Environmental Protection Agency (GB 3095–2012), we classified the PM_2.5_ concentration into five categories to show the potential health effects of each category, as shown in Table 5. We measured the potential health effects of air pollution from two perspectives: the individual-oriented and geographical space–oriented.

**Figure 11:**
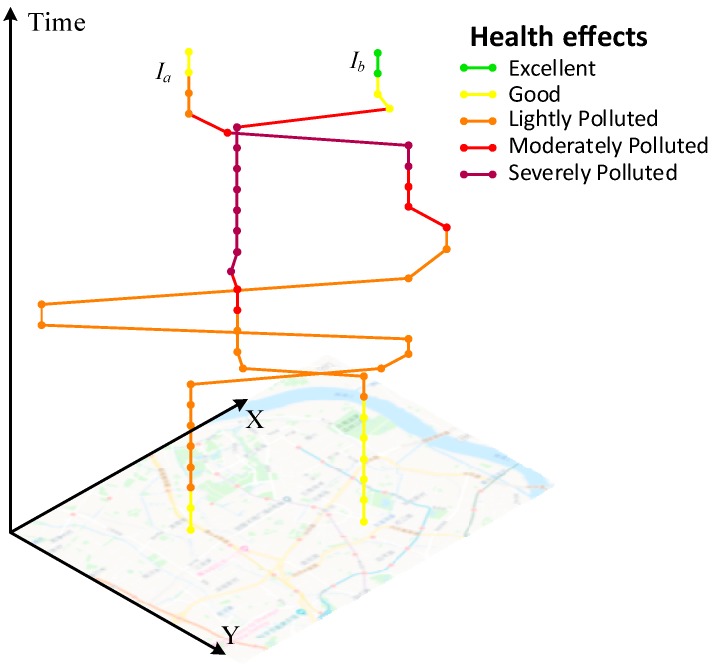
Illustration of individual exposure-level trajectories.

For the individual-oriented perspective, we selected two individuals ($I_{a}$ and $I_{b}$), whose mobilities were considerably different from each other, to show how individual movement behaviors affect exposure estimates and potential health effects during a day. The space-time paths represent the individuals’ trajectories in 3D space, and the colors of the trajectory segments represent the corresponding potential health effects [8]. As shown in Figure 11, both individuals should take appropriate precautions when spending their time outdoors. Individual $I_{b}$ has longer red-colored and purple-colored segments than does individual $I_{a}$, which means that individual $I_{b}$ experiences PM_2.5_ concentrations over moderately polluted levels for longer times. If individual $I_{b}$ belongs to the sensitive crowd, the necessary precautions should be taken or avoid outdoor activities. With our method, we can obtain fine spatiotemporal resolution of exposure-level trajectories for each individual more accurately, so as to enhance the individuals’ awareness of protection and reduce their risk of respiratory or heart diseases during trip planning.

**Figure 12:**
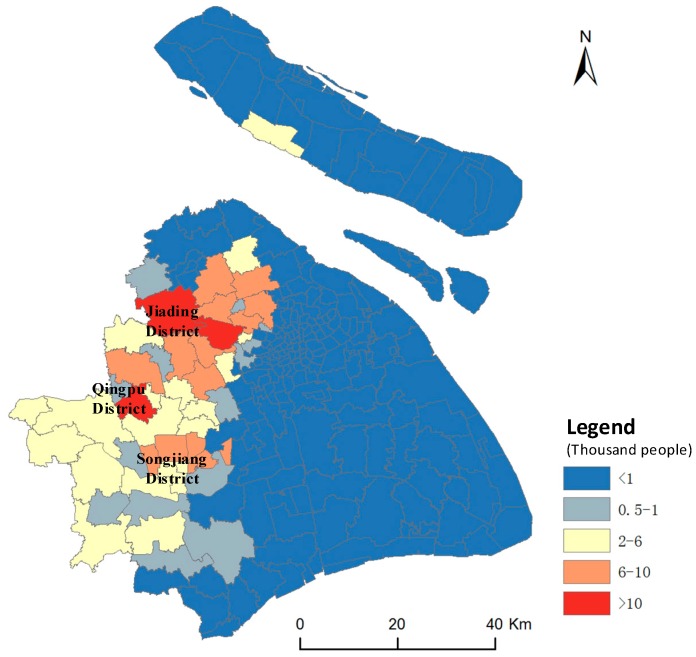
Residential locations with high exposure individuals.

As for the geographical space–oriented perspective, understanding the residences of people with high air pollution exposures can help government organizations prioritize resources and strengthen publicity purposefully to reduce the potential health effects. Therefore, we selected individuals with high exposure levels to air pollutions (whose total exposure levels during the whole week were in the top 20% of all individuals) and speculated their residential locations using their most frequently visited location during the nighttime (from 22:00 to 06:00) [72,73]. These possible residential locations of mobile phone users were aggregated to subdistrict levels, and the results are shown in Figure 12. 

**Figure 13:**
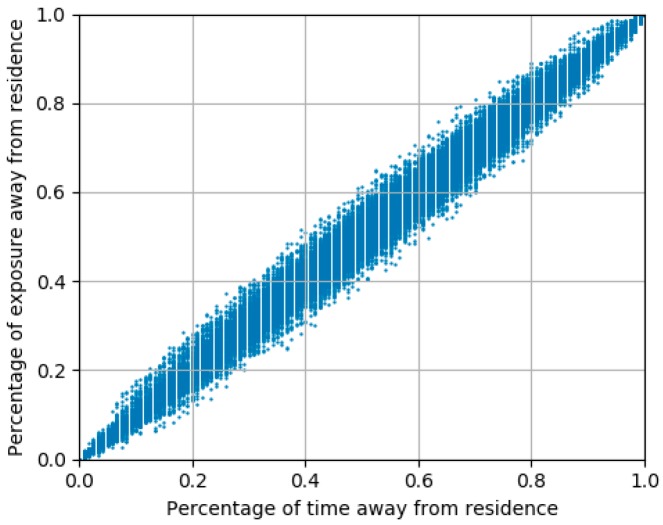
Relationship between the percentage of residents’ time away from residence and the corresponding percentage of exposure levels.

We can see that the individuals with high exposure levels mostly lived in the western part of Shanghai, and further concentrated in the Songjiang District, Jiading District, and Qingpu District. For more details, we chose the top five subdistricts in which the highest exposure individuals lived and calculated the percentage of time for which residents were away from their residences and the corresponding percentage of exposure away from residence, as shown in Figure 13. The results show that there is a positive linear relationship between the percentage of residents’ time away from their residence and the corresponding percentage of their exposure, which indicates that people with a large percentage of movement time (due to a huge potential of movements in polluted areas) tend to have large air pollution exposures, which are not only restricted to their residential areas. Table 6 shows that the residents in these areas were exposed to air pollution levels above the lightly polluted level for more than 50% of the time and were exposed to severely polluted levels for about 5% of the time, which indicated that these residents may experience irritation and the sensitive individuals among them may experience serious conditions. These results could help government organizations prioritize resources in terms of air pollution issues geographically and provided technical and theoretical support for policy-driven environmental actions.

## 5. Conclusions

This study proposed a method for estimating dynamic individual air pollution exposures using trajectories reconstructed from mobile phone data. This method mitigates the gap of spatiotemporal resolution between human movement data and air pollution monitoring data, thereby assisting in the estimation of individuals’ air pollution exposures more accurately and comprehensively at a high spatiotemporal resolution. Using the city of Shanghai as a case study, we compared three different types of exposure estimates obtained via (1) reconstructed mobile phone trajectories, (2) recorded mobile phone trajectories, and (3) residential locations. The results show that exposure estimates using reconstructed mobile phone trajectories are significantly different from the other two types of estimates, and such differences are higher when using recorded mobile phone trajectories than when using home locations for exposure estimates. This demonstrated the necessity of trajectory reconstruction in exposure and health risk assessments. Additionally, we measured the potential health effects of air pollution from both individual and geographical perspectives, which helped reveal the temporal variations in individual exposure levels and the spatial distribution of residential areas with high exposure levels.

By using the reconstructed mobile phone trajectories to measure human behaviors, this method provided a more accurate and comprehensive way of estimating dynamic individual air pollution exposure levels across space over time, which can help support policy-driven environmental actions and reduce potential health risks. In addition to the PM_2.5_ concentrations shown in the case study, the proposed method can be used to estimate individual exposure to other pollutants, such as NO_2_, SO_2_, and noise. Further, by updating individual mobile phone data and ambient pollution data, our method can be applied both to near-real-time estimates for individuals who are exposed to poor ambient air quality and the long-term effects of ambient pollution on human health, which could contribute to many crucial applications, such as disease surveillance and disaster loss assessment.

However, this study has several limitations and requires further exploration. First, the individual exposure estimation method suffered from an uncertain geographic context problem (UGCoP). Wherein, two kinds of contextual factors bring uncertainties to exposure estimates: the spatial configuration of corresponding spatial units and the timing and duration of exposure to those units [26,74,75]. For example, although we improve the spatiotemporal granularity of human movement data to the hourly level, an individual may have multiple activities in one hour, which makes the estimation counter-intuitive. Whereas, the proposed method for air pollution estimation using trajectories reconstructed from mobile phone data provides an alternative way to mitigate the influence of UGCoP. More accurate trajectory reconstruction algorithms and human movement data with better spatiotemporal resolution will be applied in this field to further improve the individual exposure estimation performance. In addition, the accuracy of air pollutant estimation is another important factor affecting the accuracy of individual exposure estimates. In the proposed method, we only adopted a GWR model to estimate air pollution concentrations by incorporating meteorological variables across space and air pollution values recorded by air quality monitoring stations. Considering the spatiotemporal coverage and resolution of meteorological observation stations, the satellite-based air pollutant concentration estimation algorithm might be helpful for improving the exposure estimate accuracy from another point of view. In addition, as the purpose of this study was to estimate large-scale dynamic individual exposures and lacking of quantifying methods for determining the effects on the microenvironment, the influence of the microenvironment (as mentioned in Equation (3)) was ignored in this study. How to consider the microenvironment effect in the proposed method is an interesting topic. We will focus on these issues in future work.

## Figures and Tables

**Table 1:** Instance of one individual’s trajectory data.

User ID	Date	Time (t)	Longitude (x)	Latitude (y)	Event Type
1EF53 *****	1	02:14:25	121.13 **	31.06 **	Regular update
1EF53 *****	1	08:15:11	121.13 **	31.02 **	Call (inbound)
1EF53 *****	1	09:17:12	121.12 **	31.02 **	Cellular handover
1EF53 *****	…	…	…	…	
1EF53 *****	7	21:13:06	121.44 **	31.08 **	Call (outbound)

Note: Accurate coordinate information and user ID were hidden with ** for privacy concern.

**Table 2:** Instance of ground-station PM_2.5_ Concentration Data.

Station ID	Day	Time (t)	Longitude (x)	Latitude (y)	PM_2.5_ Concentration (μm/m^3^)
1144A	1	00:00	121.41 **	31.16 **	43
1144A	1	01:00	121.41 **	31.16 **	49
1144A	1	02:00	121.41 **	31.16 **	52
…	…	…	…	…	
1150A	7	23:00	121.57 **	31.20 **	20

Note: Accurate coordinate information and user ID were hidden with ** for privacy concern.

**Table 3:** Instance of ground-station meteorological Data.

Station ID	Day	Time (t)	Longitude (x)	Latitude (y)	Wind Speed (m/s)	Horizontal Visibility (m)	Air Temperature (°C)
58012	1	00:00	116.65 **	34.66 **	1.5	200	−0.5
58012	1	01:00	116.65 **	34.66 **	1.5	300	−0.5
58012	1	02:00	116.65 **	34.66 **	1.7	200	−0.4
…	…	…	…	…			
58752	7	23:00	120.65 **	27.78 **	1.7	4500	8.8

Note: Accurate coordinate information and user ID were hidden with ** for privacy concern.

**Table 4:** K-S test results.

Day Type	Estimate Pairs	K-S Statistics	*p*-Value
Workday	TR-EE & REC-EE	0.0039	*p* < 0.0001
	TR-EE & SL-EE	0.0214	*p* < 0.0001
Weekend	TR-EE & REC-EE	0.0036	*p* = 0.0005
	TR-EE & SL-EE	0.0233	*p* < 0.0001

**Table 5:** PM_2.5_ concentrations and health implications.

Category	PM_2.5_	Health Implications
Excellent	<35	Without health implications.
Good	35–70	Outdoor activities normally.
Lightly Polluted	70–115	Slight irritations for healthy people and slightly impact on sensitive individuals.
Moderately Polluted	115–150	Serious conditions for sensitive individuals. The hearts and respiratory systems of healthy people may be affected.
Severely Polluted	>150	Significant impact on sensitive individuals. Healthy people will commonly show symptoms.

**Table 6:** Details of the exposure risk percentage of residents in the top five subdistricts.

Subdistrict	Excellent	Good	Lightly Polluted	Moderately Polluted	Severely Polluted
Anting County	0.94	46.03	39.43	8.56	5.04
Jiangqiao County	2.45	46.95	38.33	7.30	4.97
Xiayang Subdistrict	12.13	29.72	46.65	6.03	5.46
Huacao County	2.44	46.45	38.75	7.29	5.07
Fangsong Subdistrict	13.15	30.53	45.97	4.76	5.58
